# Lipid Profile and Aquaporin Expression under Oxidative Stress in Breast Cancer Cells of Different Malignancies

**DOI:** 10.1155/2019/2061830

**Published:** 2019-07-11

**Authors:** Claudia Rodrigues, Lidija Milkovic, Ivana Tartaro Bujak, Marko Tomljanovic, Graça Soveral, Ana Cipak Gasparovic

**Affiliations:** ^1^Research Institute for Medicines (iMed.ULisboa), Faculty of Pharmacy, Universidade de Lisboa, 1649-003 Lisboa, Portugal; ^2^Department of Biochemistry and Human Biology, Faculty of Pharmacy, Universidade de Lisboa, 1649-003 Lisboa, Portugal; ^3^Rudjer Boskovic Institute, Zagreb, Croatia

## Abstract

Breast cancer is the major cause of tumor-associated mortality in women worldwide, with prognosis depending on the early discovery of the disease and on the type of breast cancer diagnosed. Among many factors, lipids could contribute to breast cancer malignancy by participating in cellular processes. Also, aquaporins are membrane channels found aberrantly expressed in cancer tissues that were correlated with tumor aggressiveness, progression, and metastasis. However, the differences in lipid profile and aquaporin expression between cell types of different malignant potential have never been investigated. Here, we selected three breast cancer cell lines representing the three major breast cancer types (hormone positive, HER2^NEU^ positive, and triple negative) and analyzed their lipid profile and steady state lipid hydroperoxide levels to correlate with cell sensitivity to H_2_O_2_. Additionally, the expression profiles of AQP1, AQP3, and AQP5 and the Nrf2 transcription factor were evaluated, before and after oxidative challenge. We found that the lipid profile was dependent on the cell type, with the HER2-positive cells having the lowest level PUFA, whereas the triple negative showed the highest. However, in triple-negative cancer cells, a lower level of the Nrf2 may be responsible for a higher sensitivity to H_2_O_2_ challenge. Interestingly, HER2-positive cells showed the highest increase in intracellular ROS after oxidative challenge, concomitant with a significantly higher level of AQP1, AQP3, and AQP5 expression compared to the other cell types, with AQP3 always being the most expressed isoform. The AQP3 gene expression was stimulated by H_2_O_2_ treatment in hormone-positive and HER2^NEU^ cells, together with Nrf2 expression, but was downregulated in triple-negative cells that showed instead upregulation of AQP1 and AQP5. The lipid profile and AQP gene expression after oxidative challenge of these particularly aggressive cell types may represent metabolic reprogramming of cancer cells and reflect a role in adaptation to stress and therapy resistance.

## 1. Introduction

Breast cancer is the major cause of tumor-associated mortality in women worldwide. The prognosis depends on the early discovery of the disease as well as on the diagnosis. Generally, breast cancer is grouped into subtypes according to therapy, in hormone positive (estrogen- and progesterone-positive tumors) which are treated by hormones, HER2-positive (positive for the human epidermal growth factor receptor 2 (HER2)) which are treated with trastuzumab, and triple-negative tumors, which are negative for estrogen, progesterone, and HER2 receptors and are particularly aggressive, with a higher propensity for metastasis [1]. Although several biomarkers and criteria are in place to assist diagnosis and prognosis after surgery, it is imperative to find new markers and therapeutic targets and to develop new and better therapies able to bypass tumor adaptation.

Malignant cells commonly have different metabolic requirements than normal cells. Malignant cells tend to rely on glycolysis and anaerobic metabolism, require increased amount of substrates, and are highly dependent on the movement of water and ions across cell membranes for the formation of lamellipodia (invadopodia) for cell migration [2]. Clinical and preclinical studies showed that aquaporins (AQPs), membrane channel proteins that mediate fluxes of water and glycerol and in some cases hydrogen peroxide (H_2_O_2_) across cell membranes [3–7], are aberrantly expressed in cancer tissues and positively correlate with tumor aggressiveness, cancer progression, and metastasis [2, 8, 9]. Overexpression of AQPs was detected in tumor cells of different origins, being associated with tumor formation, angiogenesis, cell migration, and proliferation, suggesting that AQPs might be novel targets of diagnostic and prognostic value and their modulation could be explored for anticancer treatment [10–13]. In particular, the isoforms AQP1, AQP3, and AQP5 are abundantly expressed in diverse tumors and are involved in cell proliferation and migration by mechanisms that include changes in cellular shape and enriched ATP production during cell proliferation and differentiation [12, 14–18]. In addition, the ability of AQP3 and AQP5 to facilitate H_2_O_2_ diffusion through cell membranes [4, 7] may explain their role in activation of signaling cascades via H_2_O_2_-mediated signaling with impact in cell proliferation and resistance to oxidative stress [7, 19, 20].

A few studies have reported the expression of AQPs in breast cancer, mainly AQP1, AQP3, and AQP5, that were found higher expressed in tumors than in normal adjacent tissues [21]. While AQP3 overexpression in early breast cancer patients was shown to be associated with a worse prognosis in patients with the HER2-overexpressing phenotype [22], AQP1 and AQP5 were reported as independent prognostic markers of survival for breast cancer patients [23–25]. In addition, AQP1 overexpression can be induced by estrogen in a time- and dose-dependent fashion through activation of the estrogen response element (ERE) motif in the promoter region of the AQP1 gene, resulting in cell proliferation, migration, invasion, and tubule formation [26]. These data suggest the involvement of AQP1 in estrogen-induced angiogenesis by stimulation of endothelial cells via estrogen receptors, resulting in enhancement of tumor cell migration and invasion [15]. Similarly, the ERE motif was identified in the promoter of the AQP3 gene and can be activated to upregulate the AQP3 expression [27]. Overexpression of AQP3 was observed in ER-positive breast cancer tissues obtained from premenopausal patients when compared to those obtained from postmenopausal patients and is associated with the tumor histological grade and lymph node metastasis. The role of AQP3 in angiogenesis and invasion was suggested to involve regulation of the expression of epithelial-mesenchymal transition (EMT) molecules and reorganization of actin cytoskeleton, controlling tumor cell migration and invasion [27]. In addition, AQP3 expression was reported to affect breast cancer cell migration during metastasis via binding of the chemokine CXCL12 to its receptor CXCR4 [28]. Signaling via the CXCL12/CXCR4 axis induces Nox2 activation and extracellular H_2_O_2_ production, which is then rapidly transported intracellularly via AQP3.

Silencing AQP5 in an ER-positive breast cancer cell line (MCF7) significantly reduced cell proliferation and migration, strongly suggesting that AQP5 may play a role in the cell growth and metastasis of human breast cancer [17]. AQP5 upregulation was closely associated with cellular differentiation, lymph node invasion, and breast tumor stage [21]. A significant AQP5 upregulation found in tumors from early breast cancer patients indicates it may be used as a prognostic marker [24]. Interestingly, AQP5-mediated H_2_O_2_ diffusion was demonstrated to modulate cell survival in oxidative stress conditions [7].

H_2_O_2_ is a reactive oxygen species (ROS) that besides the recognized harmful role acts as an important signaling molecule in regulation of cellular redox homeostasis [29]. ROS-signaling is attained through reversible oxidation of thiol residues within proteins, where the intracellular levels of antioxidant molecules are crucial to maintain the redox balance. H_2_O_2_ regulates the Nrf2 transcription factor, one of the major antioxidative transcription factors located in the cytoplasm in complex with Keap1 [30]. Keap1 oxidation by H_2_O_2_ activates Nrf2 that translocates to the nucleus and starts the transcription of antioxidative genes [30]. Yet, if H_2_O_2_ overwhelms the capacity of the cell to cope with stress, oxidation of all intracellular molecules occurs. One of the most vulnerable molecules are lipids, especially their fatty acid moiety, which undergo peroxidation. Polyunsaturated fatty acids (PUFA) are a substrate for ROS and are prone to peroxidation, leaving a variety of reactive aldehydes as end products of these reactions [31]. Among these reactive aldehydes, 4-hydroxynonenal (HNE), malondialdehyde (MDA), and acrolein are the most studied. HNE was shown to modulate signaling pathways and consequently modulate cellular processes leading to proliferation, differentiation, or apoptosis [32], and interestingly, it also affects aquaporin activity [33, 34]. As the produced amount of reactive aldehydes depends on their substrate, i.e., PUFA, it is not surprising that tumor cells can change fatty acid content in order to modulate sensitivity to lipid peroxidation or as a response to the diseased state [35]. Additionally, fatty acids that are a substrate for HNE are omega6-PUFA, the ones that are substrates for prostaglandin production (e.g., arachidonic acid) [36], and therefore, modulation of the PUFA membrane content may affect important cellular signaling pathways. Moreover, decreasing the PUFA content may also affect sensitivity to oxidative stress and lipid peroxidation.

In this study, we aimed to evaluate the fatty acid content of breast cancer cell lines of different malignancies and correlate with the cell sensitivity to oxidative stress. Moreover, knowing that cell sensitivity to H_2_O_2_ largely depends on cell type and that AQPs are aberrantly expressed in many tumors, particularly in the most aggressive, the effect of H_2_O_2_ on the AQP1, AQP3, and AQP5 expression profile was investigated in breast cancer cell lines representing different breast cancer malignancies, together with their response to oxidative stress and reaction to the Nrf2 transcription factor. We hypothesize that variation of expression of specific AQPs can determine the cell sensitivity to oxidative stress and may represent a target for anticancer treatments.

## 2. Materials and Methods

### 2.1. Cell Lines and Culturing Conditions

The following breast cancer cell lines were used: MCF7 (estrogen receptor positive), SkBr3 (HER^NEU^ positive), and SUM159 (triple negative). Cells were grown in DMEM (Sigma-Aldrich) supplemented with 10% FCS (fetal calf serum, Sigma-Aldrich) in humidified atmosphere with 5% CO_2_ at 37°C.

### 2.2. Treatment

Cells were grown till they reached semiconfluency, and after trypsinization, they were counted and seeded for treatments. For cell viability and ROS assays, cells were seeded at 1 × 10^5^ cells per well in a 96-well plate, and for RNA and protein isolation, at a density of 1 × 10^6^ per well in a 6-well plate. Cells were allowed 24 h to attach and then were treated with hydrogen peroxide in a range of concentration for MTT and ROS or with 100 *μ*M H_2_O_2_ for RNA and protein isolation.

### 2.3. Cell Viability

The cell viability was measured by MTT EZ4U assay (Biomedica, Austria) according to the manufacturer's instructions. Briefly, 24 h after treatment, cells were incubated with the colorless solution, which is oxidized in mitochondria of living cells forming a yellow product, and the color intensity is measured at 450 nm, with 620 nm as a reference wavelength.

### 2.4. ROS

Intracellular ROS was measured using 2,7-dichlorodihydrofluorescein diacetate (DCFH-DA, FLUKA, USA). This nonfluorescent dye is deacetylated inside the cells and after oxidation by intracellular ROS turns fluorescent. Cells were seeded as described above and left for 24 h to attach, and then they were incubated for 1 h with DCFH-DA. After removing the excess of the dye, cells were washed once and were treated with H_2_O_2_ in a range of concentrations. Fluorescence intensity was measured for 1 h for every 10 minutes in order to follow the kinetics of H_2_O_2_ penetration to the cell.

### 2.5. RNA Analysis

All three cell lines were trypsinized, counted, and seeded at 10^6^ cells per well in a 6-well plate and were allowed for 24 hours to attach to the surface. Next day, cells were treated with 100 *μ*M H_2_O_2_ and were left for an additional 24 h, after which the cells were lysed in TRI Reagent (Invitrogen). Total RNA was isolated according to the manufacturer's instructions. The total RNA concentration was determined using a NanoDrop 1000 spectrophotometer (NanoDrop Technologies, Wilmington, DE, USA), and the quality was checked on the agarose gel. cDNA was obtained from 1 *μ*g of total RNA, and the reverse transcription was carried out with an oligo dT23 primer (Sigma-Aldrich) using a MultiScribe Reverse Transcriptase (Applied Biosystems).

Real-time PCR reactions were carried out using a CFX96 Real-Time System C1000 (Bio-Rad, Hercules, CA, USA), the TaqMan Universal PCR Master Mix (Applied Biosystems, Thermo Fisher Scientific, Waltham, MA, USA), and the following specific TaqMan predesigned gene expression primers: AQP1 (Hs01028916_m1), AQP3 (Hs01105469_g1), and AQP5 (Hs00387048_m1) (Applied Biosystems). The relative quantification of gene expression was determined using the 2^-*Δ*Ct^ method (adapted from [37]) with *β*-actin as the endogenous control. All samples were run in triplicate and the average values were calculated.

### 2.6. Fatty Acid Analysis

Cells were treated as described above, and at the end of treatment, they were trypsinized, and the dry pellet was stored at -80°C till analysis. Lipids were isolated according to modification of the Folch method [38]. Briefly, 5 ml of chloroform was added to the samples and then mixed thoroughly. An aqueous solution of MgCl_2_ (1.5 ml; 0.034%, *w*/*v*) was added to the samples, which were then vortexed and centrifuged. The upper aqueous phase was removed, a 2 M solution of KCl in methanol (2.5 ml; 4 : 1, *V*/*V*) was added, and then the samples were vortexed and centrifuged. The aqueous phase was removed, a chloroform/methanol solution (2.5 ml; 2 : 1, *V*/*V*) was added, and then the samples were vortexed and centrifuged. The hydrophobic phase was collected and transferred to a new tube, and the solvent was removed by evaporation in the nitrogen gas stream. Dry residues were stored at -80°C until further analysis.

To form fatty acid methyl esters, a 0.5 M solution of KOH in methanol [39] was added to the lipid extracts for 10 min at RT. Fatty acid methyl was then extracted with n-hexane and analyzed by gas chromatography (GC). GC analyses of total fatty acids were performed by using a Varian 450-GC equipped with a flame ionization detector. Stabilwax column (crossbond carbowax polyethylene glycol, 60 m × 0.25 mm) was used as a stationary phase with helium as a carrier gas. The heating was carried out at a temperature of 150°C for 1 min followed by an increase of 5°C/min up to 250°C. The methyl esters were identified by comparison with the retention times of commercially available standards.

### 2.7. Nrf2 Protein Analysis

Cells were grown and treated in 6-well plates as described above. For protein analysis, cells were harvested in a RIPA buffer, and protein concentration was measured according to Bradford [40]. Aliquots containing 25 *μ*g protein were separated by SDS-PAGE on 12.5% resolving gel. Proteins were transferred to the nitrocellulose membrane (Roti-NC 0.2 *μ*m; Carl Roth). The membrane was blocked with 2% nonfat dry milk, and after removing the blocking solution, the membrane was incubated with anti-Nrf2 antibody (Cell Signaling Technology). After washing, the membrane was incubated with Anti-rabbit IgG, HRP-linked Antibody (Cell Signaling Technology) (1 : 2000 dilution in blocking buffer; CST). Signal was visualized with a SuperSignal™ West Pico PLUS Chemiluminescent Substrate (TFS), and the chemiluminescence was detected using the Alliance 4.7 Digital Imaging System (UVITEC, Cambridge, UK). Signals were quantified using the ImageJ software [41].

### 2.8. Statistical Analysis

All the experiments were performed in biological and technical triplicates. Results were expressed as mean ± SEM of at least three independent experiments. Statistical analysis between groups was performed by two-way ANOVA and confirmed by the nonparametric test Mann–Whitney using the GraphPad Prism software (GraphPad Software, La Jolla, CA, USA). *p* values < 0.05 were considered statistical significant.

## 3. Results and Discussion

**Figure 1:**
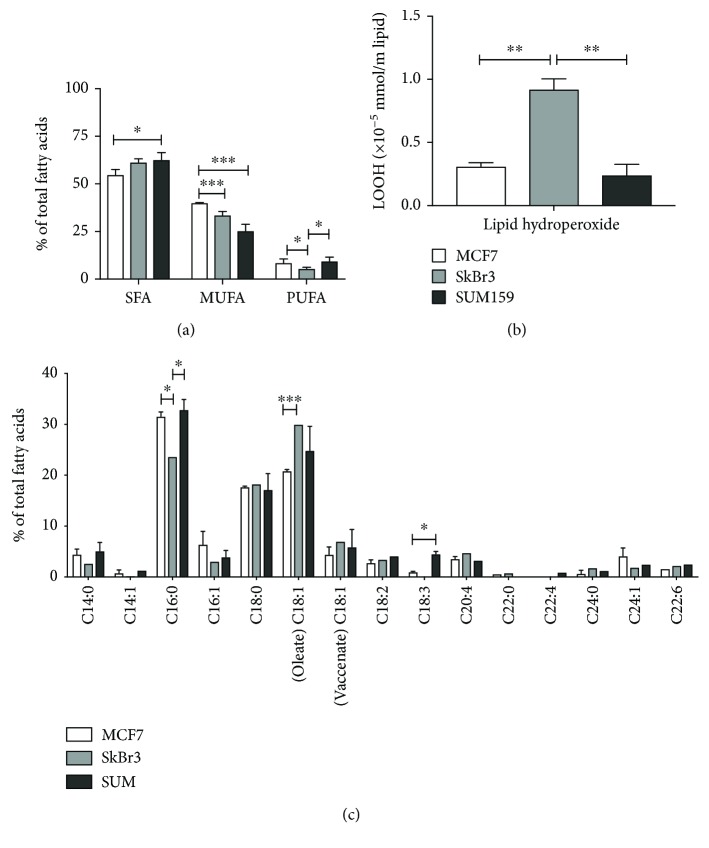
Lipid profile and steady state lipid hydroperoxide levels. (a) Composition of total fatty acids (%) of MCF7, SkBr3, and SUM159 cells. (b) Level of lipid hydroperoxide (LOOH) in MCF7, SkBr3, and SUM159 cells. (c) Specific analysis of individual fatty acids (%) in MCF7, SkBr3m and SUM159 cells. ALA: *α*-linolenic acid. Data are the mean ± SEM of three independent experiments. ^∗^*p* < 0.05, ^∗∗^*p* < 0.01, and ^∗∗∗^*p* < 0.001.

Among the risk factors for breast cancer, obesity is certainly one of the most mentioned, which correlates with the type of the cancer in pre- or postmenopausal age [42]. These data indicate the specific role of lipids in breast cancer malignancy, acting as active participants in cellular processes, such as arachidonic acid, which is a precursor for prostaglandin synthesis [36]. In addition, fatty acids, especially polyunsaturated fatty acids (PUFA), are prone to ROS attack resulting in reactive aldehyde production, which in turn acts as second messengers of free radicals [31]. In order to investigate the role of lipids/fatty acids in breast cancer malignancy, we have selected three breast cancer cell lines representing the three major breast cancer types: hormone positive (MCF7), HER2^NEU^ positive (SkBr3), and triple negative (SUM159). In these cells, we first analyzed their lipid profile and steady state lipid hydroperoxide (LOOH) levels (Figure 1).

The lipid profile showed that the MCF7 cell line has the lowest content of saturated fatty acids (SFA), while SUM159 has the highest (Figure 1(a)). The most prominent change of individual SFA is C16:0, palmitic acid, which has the lowest level in SkBr3 cells (Figure 1(c)). Palmitic acid is a signaling molecule acting through posttranslational modifications of proteins as on-off switch of protein activity. In cancer, palmitic acid enhances proliferation, metastasis, and invasiveness by stabilizing oncogenic proteins [43]. Lower levels of palmitic acid in SkBr3 cells could be due to the fact that its tumorigenity arises from overexpression of the EGF receptor (HER2^NEU^), making it hypersensitive to EGF stimulation, which is not affected by palmitoylation. The content of monounsaturated fatty acids (MUFA) showed an opposing tendency; while MCF7 had the highest levels, SUM159 had the lowest levels of MUFA. Similar to SFA, slight but not significant differences are present between most of the MUFA, with exception of the oleic acid (C18:1), which is significantly increased in SkBr3 cells compared to MCF7. Interestingly, oleic acid is decreased in cancer tissues [44], and treatment of colon cancer cell lines CaCo2 and HT29-MTX with oleic acid supported the differentiation to enterocyte and goblet cell phenotype, respectively [45]. Although HER2^NEU^-positive tumors are associated with a more aggressive phenotype, it seems that their lipid metabolism resembles more like normal cell counterparts, but this still needs further investigation.

Although elevation of SFA indicates a potential protective mechanism against oxidative stress, due to lower levels of the substrate for peroxidation, this was not accompanied by a decrease in PUFA, as there were no significant differences between the MCF7 and SUM159 cells, while SkBr3 had significantly lower levels of these fatty acids (*p* < 0.05). Yet, specific analysis of individual PUFA showed a significantly higher level of *α*-linolenic acid (ALA) in SUM159. ALA is an essential omega3-PUFA, which is used to synthesize eicosapentaenoic acid (EPA, 20:5n-3) and docosahexaenoic acid (DHA, 22:6n-3). ALA is associated with antitumorigenic effects in mice fed with an ALA-rich diet [46] and was reported to be toxic for MCF7 cells causing cell cycle arrest and apoptosis through mitochondrial membrane depolarization [47]. In MCF7 cells, it was shown that exogenously added ALA decreased growth via estrogen receptor-mediated signaling [48]. No doubt that in cancer cells, metabolic reprogramming occurs and is one of the hallmarks of cancer. This reprogramming also includes reprogramming of the lipid metabolism, but it is still uncertain if the changes are due to fatty acid uptake or de novo synthesis [49]. It was shown that fatty acid content changed in the red blood cell membrane in cancer patients [50], indicating that fatty acid content is involved in cancer growth/development, but the precise role is not clarified. In the context of ALA, the differences between cell types of different malignant potential have never been investigated.

The slight differences observed in MUFA and PUFA content in the three tested cell lines prompted us to additionally measure their lipid hydroperoxides (LOOH). LOOH are intermediate products in peroxidation, and this is the step that can multiply the lipid peroxidation cascade. Therefore, we investigated if the slight and nonsignificant differences between the cell lines could be multiplied in this reaction step. These results showed that SkBr3 cells, a HER2^NEU^-positive cell line, had significantly higher content of steady state LOOH (Figure 1(b)). Since this unexpected result did not correlate with the lower PUFA content in SkBr3 (Figure 1(a)), we proceeded to investigate if the observed differences in fatty acid content and LOOH levels in the steady state could influence cell sensitivity to H_2_O_2_.

**Figure 2:**
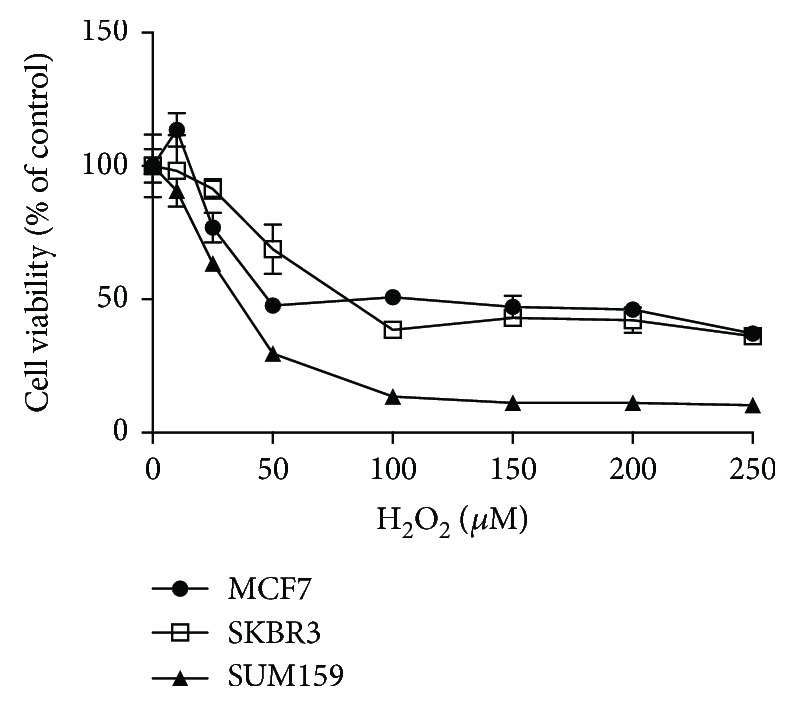
Cell viability upon oxidative stress. Cell viability of MCF7, SkBr3, and SUM159 cell lines were determined by MTT assay after exposure to a range of H_2_O_2_ concentrations for 24 h. Data are the mean ± SEM of three independent experiments.

Thus, we evaluated cell viability upon H_2_O_2_ challenge by MTT assay (Figure 2). H_2_O_2_ is a small molecule produced intracellularly under physiological conditions in a controlled, as well as uncontrolled, manner. When H_2_O_2_ is produced by the cell itself, in physiological conditions, it contributes to redox signaling [29]. Interestingly, the hormone-positive MCF7 and the HER2^NEU^-positive SkBr3 cell lines showed similar sensitivity, while the triple-negative line SUM159 was more sensitive to H_2_O_2_ challenge. This difference in sensitivity cannot be explained by the fatty acid content analysis, where SUM159 and SkBr3 cells show similar total SFA content (Figure 1(a)), which is observed in almost every individual SFA with exception of C16:0 (Figure 1(c)). SFA do not contain double bonds that are substrates for ROS attack and are therefore more resistant to peroxidation [51]. As a consequence, it is supposed that cells with higher SFA content could be more resistant to oxidative stressors. However, this is not entirely true, as evidenced by the SkBr3 cell line, which had the highest LOOH content but showed higher resistance to H_2_O_2_ than SUM159. A possible explanation is that SkBr3 cells may have an adequate antioxidative defense to cope with the (per)oxidation challenge.

As PUFA content and H_2_O_2_ sensitivity did not show correlation, we then investigated if differences in H_2_O_2_ sensitivity were due to differences in H_2_O_2_ influx between these cell lines. As the viability curves showed a plateau at 100 *μ*M H_2_O_2_ and above, this was the highest concentration used for influx measurements. According to the eustress theory, an extracellular concentration of 100 *μ*M H_2_O_2_ corresponds to intracellular 1 *μ*M H_2_O_2_, which may trigger both effects, oxidative eustress and distress [52]. Oxidative eustress is a term for positive low levels of oxidative stress, turning on adaptive mechanisms without damaging the cells. On the other hand, distress refers to higher levels of stress leading to pathological adaptation with cellular damage resulting in different diseases [52]. Which of these effects will occur in a certain cell line/type depends on the cell gene expression profile of the antioxidative defense system but also on energy metabolism; in addition, the expression of AQPs in cell membranes fine-tuning H_2_O_2_ fluxes may also dictate the cell response to oxidative stress.

**Figure 3:**
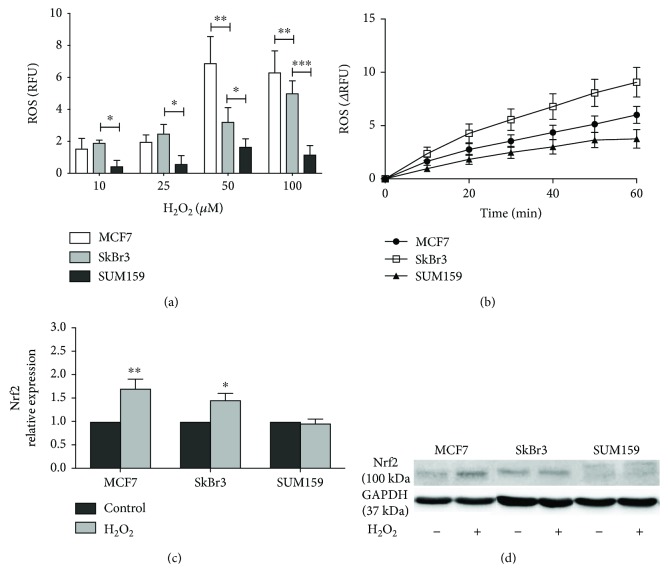
Antioxidant defense response upon oxidative stress. (a) ROS intracellular accumulation 60 minutes after H_2_O_2_ treatment. Cells were incubated with a range of H_2_O_2_ concentrations (10 *μ*M to 100 *μ*M), and ROS accumulation was recorded over 60 min. Data was normalized with basal levels of intracellular ROS. (b) Representative signals of fluorescence output after addition of 100 *μ*M H_2_O_2_. (c) Nrf2 protein expression of treated (100 *μ*M H_2_O_2_) vs. nontreated cells, normalized to GAPDH. (d) Nrf2 protein expression of cells treated (+) and nontreated (-) with 100 *μ*M H_2_O_2_ (representative blots). ^∗^*p* < 0.05, ^∗∗^*p* < 0.01, and ^∗∗∗^*p* < 0.001.

Therefore, we first analyzed the levels of ROS accumulation after 60 minutes of H_2_O_2_ treatment in the three cell lines (Figure 3(a)). As expected, the most pronounced differences between ROS levels were found when cells were treated with 100 *μ*M H_2_O_2_. Since this concentration induced a significantly different level of ROS between the three cell lines and was the maximal inhibitory concentration of cell viability after 24 h (Figure 2), we followed the rate of ROS accumulation after a challenge with 100 *μ*M H_2_O_2_. Interestingly, SkBr3 cells showed the highest increase in intracellular ROS, and SUM159 had the lowest, indicating that this is not the only factor that accounts for the difference in sensitivity.

Considering that the antioxidative defense system may be in part responsible for cell resistance to oxidative stress, we then analyzed the expression of the antioxidant transcription factor Nrf2 under oxidative stress conditions (Figures 3(c) and 3(d)). As expected, after exposure to H_2_O_2_, the cell lines MCF7 and SkBr3 increased their Nrf2 levels, in line with the Nrf2 role in the state of the disturbed cellular redox balance [30]. On the other hand, for the triple-negative cell line, SUM159 had levels of Nrf2 lower than those of MCF7 and SkBr3 that were not changed upon H_2_O_2_ treatment. The lower levels of Nrf2 and the lack of reactivity to H_2_O_2_ may explain the higher sensitivity to H_2_O_2_ of SUM159 cells. Nrf2 targets a variety of antioxidative defense genes including glutamate-cysteine ligase, catalytic subunit and modifier subunit (GSH synthesis), thioredoxin reductase 1, peroxiredoxin 1, Nqo1, and glutathione-S-transferase family [53], meaning that low levels of Nrf2 consequentially lead to low levels of antioxidative defense. The failure to increase Nrf2 levels after H_2_O_2_ challenge may be due to the inability of its steady state inhibitor Keap1 to release Nrf2 upon sensing oxidative conditions. When cells are not under oxidative stress, Nrf2 is in complex with Keap1-Cul3-ubiquitinase E2 complex and is being ubiquitinated for subsequent peroxisomal degradation [30]. In stress conditions, Keap1 cysteine moieties are oxidized by the stressor, leading to release of Nrf2 and activation of the antioxidative gene transcription. In SUM159 cells under oxidative stress, lack of Nrf2 response indicates that this pathway is probably downregulated, causing increased sensitivity. This result can be considered quite a paradox, as one would expect that aggressive cancer cells are more resistant to stress. Still, all three cell lines reach plateau at 100 *μ*M H_2_O_2_, with the lowest value for SUM159. Tumor cells are followed by a paradox about the role of the ROS and the use of antioxidants. ROS are one of the factors in cancer development, as well as factors in cancer therapy. Antioxidants are a factor in cancer prevention but also a factor which will support tumor growth and counteract tumor therapy [54]. In this context, prolonged exposure to oxidative stress could provide an answer in the processes underlying this initial sensitivity as the aggressiveness is referred to survival of the small subpopulation of cancer cells which can be activated under certain conditions and cause recurrence of the disease. Supporting the paradox of oxidative stress/antioxidants is the role of Nrf2 in carcinogenesis. Nrf2 is considered as a protective factor in the early stage and a resistance factor in the late stages in cancer development [30]. Although Nrf2 levels are elevated in certain types of cancer [30, 55] thereby providing protection against ROS, its levels in normal tissues are fluctuating and are regulated by balance between its synthesis and degradation [56].

**Figure 4:**
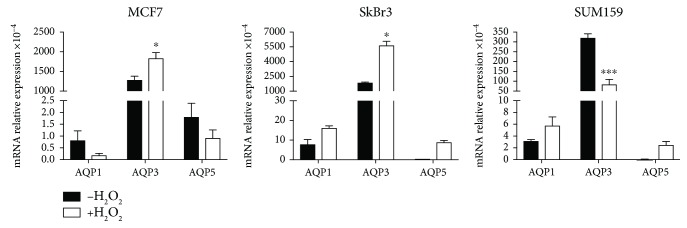
Effect of H_2_O_2_ treatment on aquaporin gene expression. AQP1, AQP3, and AQP5 mRNA expression was determined after treating cells with 100 *μ*M H_2_O_2_ for 24 h. Data represent mean ± SEM from three independent experiments. ^∗^*p* < 0.05 and ^∗∗∗^*p* < 0.001 versus nontreated cells.

In order to investigate the influence of H_2_O_2_ influx on the three breast cancer cell lines, the expression profile of the three AQP isoforms most expressed in breast cancer tissues, namely, AQP1, AQP3, and AQP5 [57], was investigated. The expression analysis revealed that the level of expression of these AQPs is significantly different between the three cell lines, being much higher in the HER2^NEU^-positive cell line SkBr3 (Figure 4). The lower basal levels of AQPs expressed in SUM159 cells compared to the other tested cell lines may not be easy to understand. It is well accepted that AQPs are overexpressed in cancer tissues compared to their normal counterparts and that the overexpression pattern follows tumor aggressiveness. It is worth mentioning that the tested cell lines MCF7, SkBr3, and SUM159, although representative of an increasing level of malignancy, do not derive from the same primary tumor specimen nor have the same degree of differentiation. Knowing that AQPs are tissue- and cell type-specific, and that breast cancer is highly heterogeneous, it is expectable that their basal gene expression level is not comparable in these three cell lines. In addition, the low levels or absence of HER2 receptors characteristic of triple-negative breast cancer cells such as SUM159 may also correlate with low levels of other membrane proteins such as AQPs. Moreover, the weak basal Nrf2 expression found in these cells may imply the existence of a complementary protective mechanism to cope with oxidative challenges.

In all the cell lines, AQP3 was the aquaporin most expressed, followed by lower levels of AQP1 and AQP5. This result is not surprising, since AQP3 has been reported as an important player in cancer biology and its expression is correlated with the epidermal growth factor receptor (EGFR) in several types of cancer [58].

Interestingly, AQP3 expression was stimulated by H_2_O_2_ treatment in MCF7 and in SkBr3 cells and followed the pattern of Nrf2 expression. Yet, unlike MCF7 and SkBr3 cells, in SUM159 cells, H_2_O_2_ treatment downregulated AQP3 expression, which together with the nonresponsiveness of Nrf2 activation indicates that these cells are intrinsically less responsive to external oxidative insults and are more prone to treatment resistance, corroborating their malignant aggressiveness and metastatic potential.

The mechanism by which AQP3 contributes to cancer malignancy could be due to regulation of H_2_O_2_ influx, which is enhanced after CXCL12 stimulation in MCF7 cells [28]. A previous study with the epidermoid carcinoma cell line A-431 showed that AQP3 coprecipitated and colocalized with EGFR, and its regulation of H_2_O_2_ influx was imperative for EGF/EGFR signaling, including Erk and Akt activation [19]. Our results show that SkBr3, the HER2^NEU^-positive cell line, had the highest level of AQP3 (Figure 4). Interestingly, recent studies analyzing a panel of breast and ovarian cancer cell lines [59, 60] showed that SKBR3 cells express the highest levels of HER2 among all detected cell lines [60], supporting the above reported relation between EGFR, one of the most studied HER family receptors and a key oncogenic driver in many epithelial cancers including breast cancer [61], and AQP3 expression. In addition, the fact that AQP3 also transports glycerol that can be used as an energy source in the glycolytic pathway or as a building unit for phospholipids, thus contributing to cancer growth, cannot be disregarded.

Regarding AQP1 and AQP5, while in the least malignant cell line MCF7 their expression decreased after H_2_O_2_ treatment, the opposite is observed for the more aggressive SkBr3 and SUM159 cells. The upregulation of AQP gene expression after H_2_O_2_ challenge in these particularly aggressive cell types may reflect their role in adaptation to stress further contributing to therapy resistance.

In conclusion, breast cancer cells with different malignancies show distinct lipid steady state profiles and sensitivity to oxidative stress. Their altered pattern of aquaporin gene expression triggered by oxidative stress brings evidence to the involvement of these membrane proteins in cancer aggressiveness. Both aquaporins and Nrf2 are important players in the regulation of normal cellular homeostasis, while their elevated levels in tumors represent critical factors, which could contribute to malignancy and therapy resistance. The interplay of cellular antioxidative defense factors such as Nrf2 with specific AQPs in the progression of breast cancer deserves further investigation.

## Data Availability

All the data used to support the findings of this study are included within the article.
